# Heat Stress, Indoor Microclimate, and Lactation Feed Intake in Commercial Mediterranean Sows: Parity Effects and a Daily Resolution Predictive Model

**DOI:** 10.3390/life16081306

**Published:** 2026-08-09

**Authors:** Lampros Fotos, Aris Pourlis, Eirini Valasi, Georgios I. Papakonstantinou, Konstantinos Kousenidis, Athanasios Sougias, George Tsegas, Eleftherios Chourdakis, Zisis Tsiropoulos, Alexandra V. Michailidou, Christos Vlachocostas, Vasileios G. Papatsiros

**Affiliations:** 1 Clinic of Medicine, Faculty of Veterinary Medicine, University of Thessaly, 43100 Karditsa, Greece; lfotos@uth.gr (L.F.); geopapak@vet.uth.gr (G.I.P.); 2 Laboratory of Anatomy, Histology and Embryology, Faculty of Veterinary Medicine, University of Thessaly, 43100 Karditsa, Greece; apourlis@uth.gr; 3 Laboratory of Physiology, Faculty of Veterinary Science, University of Thessaly, 43100 Karditsa, Greece; evalasi@uth.gr; 4 Department of Agriculture, School of Geosciences, International Hellenic University, 57400 Thessaloniki, Greece; kousenidis@ihu.gr; 5 Nuevo, 32009 Schimatari, Greece; th.sougias@nuevo-group.com; 6 Sustainability Engineering Laboratory, Aristotle University, 54124 Thessaloniki, Greece; gtsegas@auth.gr (G.T.); chourdakis@auth.gr (E.C.); amicha@meng.auth.gr (A.V.M.); vlahoco@meng.auth.gr (C.V.); 7 Department of Agrotechnology, School of Agricultural Sciences, University of Thessaly, 41500 Larissa, Greece; ztsiropoulos@uth.gr

**Keywords:** swine production, Temperature–Humidity Index (THI), thermal stress, environmental monitoring, Mediterranean, Precision Livestock Farming (PLF)

## Abstract

Heat stress (HS) suppresses voluntary feed intake in lactating sows, yet individual-level characterization of indoor and outdoor Temperature–Humidity Index (THI) linked to feed intake is rare in Mediterranean commercial settings. This prospective observational study enrolled 272 F1-crossbred sows (parities 1–7) farrowing from May–October 2025 at a commercial Greek farm. Outdoor THI was computed from 8760 hourly records and indoor THI from calibrated dataloggers; individual daily lactation feed intake (days 1–30) was recorded via RFID transponder-feeders. Building cooling (ΔTHI = 3.7–14.3) attenuated but did not eliminate mild-to-moderate indoor stress from June–September. Only parity and stillbirth count were Bonferroni-significant correlates of feed intake (parity: β = +0.142, *p* = 0.002). A sow-day Gradient Boosting model, evaluated by grouped cross-validation to prevent within-sow leakage, achieved CV-R^2^ = 0.406 ± 0.045, providing a validated, herd-tested framework for parity-stratified feeding management, pending external validation across farms, genetics, and climates.

## 1. Introduction

Lactating sows represent the most thermally vulnerable category within the modern farrow-to-finish production system. The thermal challenge of lactation is twofold: on the one hand, the high metabolic activity required to sustain milk production generates a substantial additional heat load—estimated at 2500–3500 kJ/d above basal metabolism—that must be dissipated to maintain body temperature homeostasis [1,2]. Moreover, the compactness of the commercial farrowing room, with several crated sows and the provision of heated creep areas for newborns, further inhibits convection and radiation in the process of thermal regulation. In cases where ambient temperature rises above the animal’s upper critical point (20–22 °C for multiparous females) [3], the predominant regulatory mechanism in the process is the lowering of voluntary feed intake, resulting in lowered heat production from digestion and leading to a lack of energy for milk synthesis and maternal weight maintenance.

Heat stress (HS) ranks as one of the most economically important environmental issues encountered in pig production. Lactating sows suffer from HS more than any other animal in pig farms because of a high rate of metabolism and hence heat production in making milk, low sweating ability, and an upper critical point of around 18–20 degrees centigrade for the thermoneutral zone [4,5]. Above that point, the sows limit their feed consumption to reduce heat production. THI (Temperature–Humidity Index) is calculated using the following equation: THI = 0.8T + RH × (T − 14.4) + 46.4 [6]. The categories of stress, as determined by the THI, include no stress (<68), low stress (68–72), moderate stress (72–78), high stress (78–89), and extreme stress (>89) [7,8]. Modern farrowing systems incorporate mechanical ventilation and evaporative cooling to alleviate HS caused by extreme outdoor temperatures. Assessing the difference between outdoor and indoor THI values (ΔTHI) and the effect of ΔTHI on individual sow feed consumption while accounting for parity and litter weight distribution remains an important but underexplored topic in the literature.

Precision Livestock Farming (PLF) has emerged as a framework for integrating continuous environmental monitoring with individual animal performance data to enable proactive, data-driven management decisions [9,10]. In pig production, PLF applications have been proposed for feed intake optimization [11], disease early warning, and reproductive management; however, decision-support tools that explicitly link real-time thermal indices to predicted lactational feed intake—with sufficient operational simplicity for direct farm deployment—remain largely absent from both the research literature and commercial software. The present study addresses this gap by combining a six-month field dataset with the development and implementation of a THI-based feed intake prediction model as a browser-based decision-support application.

Thessaly, one of Greece’s main agricultural regions, combines extensive lowland plains with a high density of livestock, making it particularly vulnerable to climate-driven HS. Based on our recent spatial distribution of maximum summer temperatures, using ERA5-reanalysis data, the livestock systems are affected by HS in the Thessaly Region over the past several years (2020–2025) [12]. HS in livestock operations in the Thessaly region is not an episodic phenomenon but a continuous and spatially organized environmental factor affecting these operations [12].

The present study aimed to (i) characterize full-year outdoor THI (mean ± SD) and indoor THI from May to October; (ii) quantify the outdoor–indoor THI correlation and ΔTHI; (iii) describe individual sow lactation feed intake (days 1–30) by unique ear-tag linkage with explicit accounting for litter balancing; (iv) identify predictors of mean daily feed intake using Bonferroni-corrected correlations, VIF-validated regression, and ANCOVA controlling for parity; and (v) develop a sow-day resolution feed intake model and evaluate, through properly grouped cross-validation, whether restructuring the outcome at this finer temporal resolution improves individual-level predictive performance.

## 2. Materials and Methods

### 2.1. Study Farm and Animal Management

The study was conducted in accordance with the Code of Practice for the Conduct of Clinical Trials for Veterinary Medicinal Products [13] and the Guide for the Care and Use of Agricultural Animals in Research and Teaching [14]. The trial protocol was reviewed and approved by the Research Ethics Committee of the University of Thessaly, Greece (Approval No. 45, dated 12 May 2025).

The experiment was carried out at a commercial farrow-to-finish pig farm situated in Thessaly, Greece (coordinates: 39°29′24.5″ N 22°21′04.7″ E, climatic zone: Mediterranean continental climate, with very warm summers and mild winters). The farm utilizes a three-week batch farrowing cycle with the gestation and lactation phases housed in different buildings. Sows in the farrowing facility are provided with farrowing stalls containing automatic ad libitum feeding systems. Six months of the experimental period started on the 1st of May and ended on the 31st of October 2025.

The farrowing building measured 60 m × 12 m (gable roof, height 3.2 m at ridge), constructed with concrete block walls (20 cm, internally insulated with 5 cm expanded polyurethane panels) and a sealed concrete floor. The building housed 64 farrowing crates (2.2 m × 0.65 m per crate, with adjacent piglet creep areas heated to 32–34 °C via electric infrared lamps). The farrowing house included 8 rooms with 8 farrowing crates per room and was housed in environmentally controlled facilities equipped with an evaporative cooling system and automated climate-control and ventilation management (BlueControl Climate, SKOV A/S, Glyngøre/Roslev, Denmark). On day 109 of gestation (approximately 7 days before the expected farrowing date), sows were transferred to individual farrowing crates (approximately 2.5 m × 2.0 m). Each crate consisted of a concrete floor for the sow and a plastic-slatted creep area for piglets. The piglet creep area provided approximately 2 m^2^ of space and was equipped with a heated creep box containing a 200 W infrared lamp to maintain temperatures between 33 and 35 °C throughout the suckling period. Fresh water was available ad libitum to all sows through nipple drinkers during lactation. Eight ventilation inlets (one per farrowing room) were distributed along the lateral walls; eight axial exhaust fans (one per farrowing room and each with a capacity of 6000 m^3^/h) were mounted on the roof ridge. The farrowing house is mechanically ventilated with axial fans mounted on the roof and controlled by a thermostat set to maintain room temperatures at 22–24 °C—a design target achievable under mild outdoor conditions (Supplementary Files S1 and S2). It is acknowledged that this target was not met during June–September 2025, when outdoor temperatures regularly exceeded 35 °C. No evaporative cooling pads or refrigerative systems were operational during the study period; mechanical ventilation was the sole thermal-attenuation mechanism. Throughout this manuscript, the term ‘building cooling’ refers to passive thermal buffering achieved by this ventilation system, not to active refrigerative or evaporative cooling.

Selection criteria for inclusion: sows farrowing during the study and with >3 days of feed history, as well as a unique identifier tag number. In all, 272 sows participated in the study: 263 F1 hybrid animals (commercial proprietary cross between hyperprolific breed dams Large White (LW) × Landrace (L) and purebred terminal Duroc boars) and 9 LW sows. Their parities varied between 1 and 7 (average 2.99 ± 1.56; P1:57, P2:70, P3:38, P4:48, P5:45, P6:13, P7:1). Each sow was uniquely identified by ear-tag number, which served as the primary data linkage key across all datasets.

A sensitivity analysis excluding the 9 LW sows confirmed a negligible breed effect on mean daily feed intake (LW: 5.065 ± 1.064 vs. F1: 5.294 ± 1.196 kg/day; t = 0.567, *p* = 0.571); all analyses include the full cohort. Three sows with lactation length = 0 days (periparturient deaths) were excluded from feed intake analyses. Five sows with lactation lengths > 30 days had feed data truncated at day 30. Individual sow live weight, body condition score (BCS), and water intake data were not available from the farm management system and are therefore absent from the prediction model. These represent priority variables for future model development (see Section 4.5).

### 2.2. Litter Balancing Protocol—Farrowing Batch Records

Cross-fostering is the relocation of piglets from their biological mother to a foster sow to equalize litter size and reduce mortality. At each farrowing, litter balancing targeted 12 weaned piglets per sow via cross-fostering and artificial nursing. The net fostering adjustment per sow was recorded as positive = received piglets and negative = donated piglets. Of 269 sows (feed analysis cohort), 215 (79.9%) donated (mean −3.0 ± 3.9; range from −15 to −1), 39 (14.3%) received (mean +3.3 ± 1.9; range from +1 to +9), and 15 (5.5%) required no adjustment. Litter size was adjusted within 24–48 h after birth, considering the functional teats of each sow. The protocol achieved 12 weaned piglets in 269/272 sows (96.7%).

Farrowing records were extracted from the farm management software for the same period. Variables included: total born piglets, born alive piglets, stillborn piglets, mummified fetuses, fostering adjustment (piglets), effectively nursed (after balancing), weaned (piglets), lactation length (days), parity, and gestation length (days).

### 2.3. Lactation Feed Intake Recording

Lactating sows were fed a commercial lactation diet (crude protein 17.5%, lysine 1.05%, metabolizable energy 13.8 MJ/kg) via an automated GESTAL Solo+ feeding system (JYGA Technologies, St-Lambert-de-Lauzon, QC, Canada) (Supplementary File S2). The GESTAL Solo+ is an ad libitum wet-dry feeder that uses RFID ear-tag technology to identify each sow and record cumulative feed consumption throughout the lactation period. The system was programmed to allow feed access at any time of day, up to a maximum of six feeding bouts per day. Feed delivery was volumetrically calibrated for each farrowing group. Mean daily feed intake (kg/day) was defined as total feed consumed divided by lactation days, computed from day 1 (farrowing) through day 30 or weaning, whichever occurred first.

### 2.4. Outdoor THI Computation

Hourly outdoor temperature (°C) and relative humidity (decimal fraction, constrained 0–1) were recorded by a Wireless Digital Weather Station Agenso MeteoIo T 2100S 4G (Agenso, Athens, Greece) throughout 2025 (*n* = 8760 hourly observations) (Supplementary File S3).

Saturation vapor pressure was computed from the Magnus equation:

*e_s_*(*T*) = 6.112 × *exp*(17.62 *T*/(243.12 + *T*))

*RH* = *e_s_*(*T_d*)/*e_s_*(*T*)

where *T* is dry-bulb and *T_d* is dew-point temperature (°C). The Temperature–Humidity Index was then calculated hourly as follows:

*THI* = 0.8 *T* + *RH* × (*T* − 14.4) + 46.4

Consistent with the NRC (1971) [15] formulation, it is acknowledged that this formula was originally derived for dairy cattle [6,15] and that no validated pig-specific *THI* formula currently exists in the literature—a gap that future research should address. Its use here follows the established convention in Mediterranean swine HS research [12,16]. and the thresholds applied by Papatsiros et al. (2026) [12]. Four HS classes were assigned: no stress (*THI* < 68), mild (68–72), moderate (72–78), high (78–82) and severe (>82). Daily and monthly summary statistics (mean ± SD, daily maximum) were computed; days with daily maximum *THI* > 72 were counted as HS days. Daily maximum *THI* values were derived from the hourly time series for each day and used to identify HS days and extreme exposure conditions. Monthly statistics were subsequently calculated based on these daily aggregates.

### 2.5. Indoor Microclimate Monitoring

Indoor farrowing-house microclimate was recorded using Kestrel 5400 Heat Stress Tracker dataloggers (Nielsen-Kellerman, Boothwyn, PA, USA; accuracy: ±0.5 °C temperature, ±2% RH) (Supplementary File S3). They were deployed at sow-height positions distributed across the rooms of the farrowing house, recording 1 h interval data from May to October 2025 (*n* = 4431 valid hourly records after exclusion of 209 records flagged during calibration checks). All eight dataloggers were factory-calibrated before deployment and cross-checked against a reference probe at the start of the study period; units deviating more than ±1 °C or ±3% RH were recalibrated. Excluded records were distributed across all sensors (mean 26 records/sensor; maximum 38 for the outlet sensor). Indoor THI at each hour was computed as the arithmetic mean of the eight sensor readings before applying the Thom (1959) [6] formula. Indoor monitoring was limited from May–October because outdoor THI remained below the no-stress threshold (<68) throughout November–April 2025, and building thermal buffering under cool conditions was not a primary study objective.

### 2.6. Thermal Loading Index (TLI)

A complementary metric—the Thermal Loading Index (TLI = indoor THI − 68, set to 0 when indoor THI < 68)—was also computed to quantify the absolute thermal burden experienced by the sow above the no-stress threshold. Both metrics are reported in Table 1 and discussed in Section 4.1. In the study cohort, TLI ranged from 0 in May and October (indoor THI below the no-stress threshold) to 7.25 in July (indoor THI = 75.25 − 68 = 7.25 units above threshold), with intermediate values in June (4.35), August (4.81), and September (0.90).

### 2.7. Statistical Analysis

Continuous data are summarized as mean ± SD. Pearson correlations between mean daily feed intake and 10 candidate predictors were computed; Bonferroni correction was applied (α/*n* = 0.05/10 = 0.005), and both raw and corrected *p*-values are reported. THI–feed correlations are reported at two levels: (a) individual sow level (*n* = 271, illustrative only; THI is a batch-level variable with 6 unique monthly values, df = 4) and (b) the correct monthly-aggregate level (*n* = 6 monthly means, df = 4). One-way ANOVA for month and parity effects on feed intake was followed by Tukey’s HSD pairwise comparisons. ANCOVA with parity as covariate assessed the month effect independently of batch parity composition. For the fostering ANOVA (k = 3 groups), post hoc power was computed for the smallest group (*n* = 15) assuming 80% power, α = 0.05, and Cohen’s f = 0.25. Normality of continuous response variables was assessed using the Shapiro–Wilk test. Mean daily feed intake did not significantly deviate from normality (W = 0.989, *p* = 0.072). Lactation length was mildly non-normal (W = 0.961, *p* < 0.001); Spearman ρ yielded equivalent conclusions to Pearson r and is reported alongside where relevant. Parity was entered as a numerical covariate in ANCOVA solely to adjust for batch parity composition (its known linear confound on month-level feed comparisons), not as a continuous predictor; this use is standard in GLM covariate adjustment (ANCOVA with discrete covariate). Although parity is recorded as discrete integer values (P1–P7), it was entered as a numeric (rather than categorical/factor) covariate because it has a natural biological ordering and an expected monotonic relationship with feed intake (increasing from primiparous to multiparous sows); modeling it as a single linear term uses one degree of freedom to capture this trend, which is standard practice for ordinal covariates in animal science ANCOVA and is distinct from parity’s use as a categorical factor in the one-way ANOVA/Tukey’s HSD analyses of Section 3.6, where the full set of pairwise group differences was of direct interest. A parity × month interaction term was also tested (two-way ANOVA): F = 1.24, *p* = 0.247, non-significant, consistent with limited power (many cells *n* < 5). Five-fold cross-validation used KFold (n_splits = 5, shuffle = True, random_state = 42); CV-R^2^ is the mean of five held-out R^2^ values. Residual analysis confirmed no heteroscedasticity (Breusch–Pagan *p* = 0.21) and 12 observations with Cook’s distance > 4/*n* (none exceeded 1.0; results robust to their exclusion). The OLS approach was maintained as the most interpretable one in this exploratory analysis; the criterion for using it in individual-level settings would be a CV-R^2^ value of ≥0.10. The Bonferroni method entails the comparison of *p*-values against 0.005 (equivalent to the multiplication of adjusted *p*-values by *n* against 0.05); both uncorrected and Bonferroni-corrected *p*-values are presented. All statistical tests were conducted in Python 3.12 (scipy 1.14, statsmodels 0.14, scikit-learn 1.5).

## 3. Results

### 3.1. Full-Year Outdoor THI Profile

**Figure 1:**
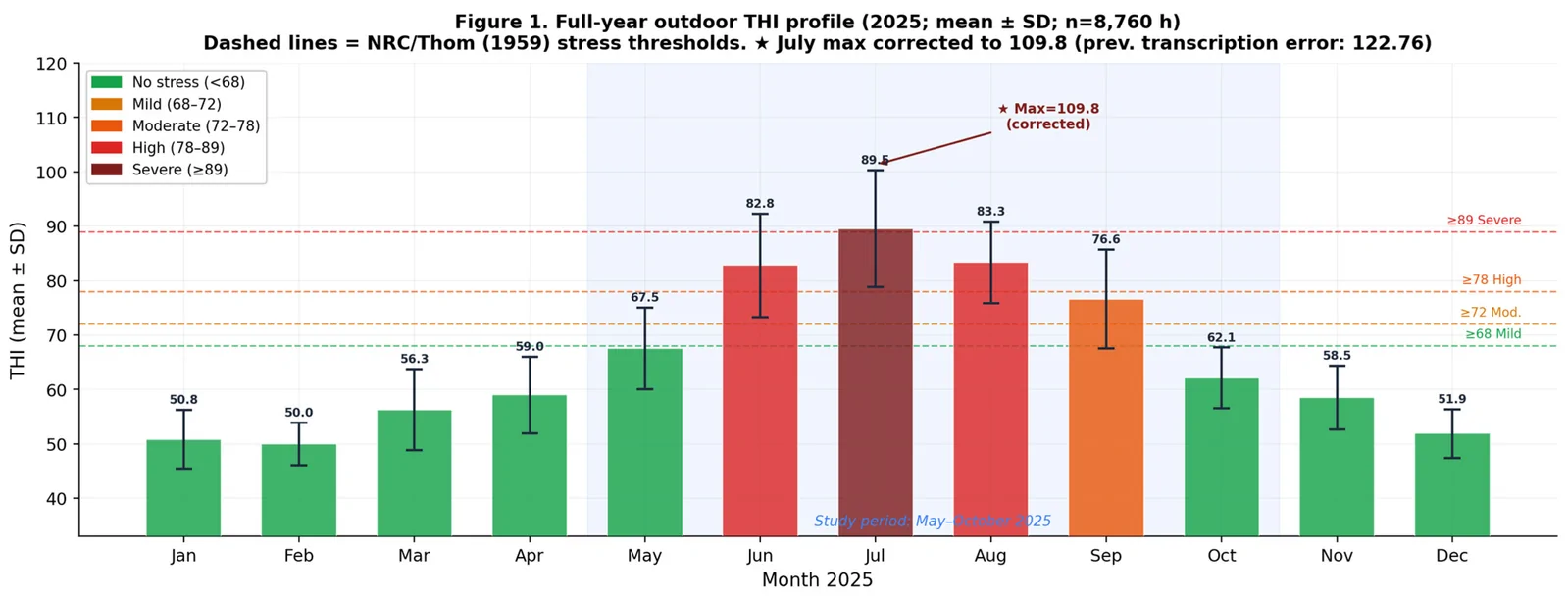
Full-year outdoor THI profile (2025; mean ± SD). Error bars = ±1 SD; dashed lines = [6,11] stress thresholds. Shaded area = study period (May–October).

Table 1 and Figure 1 show the monthly outdoor THI profile (mean ± standard deviation) for the entire year of 2025. During winter, THI was neutral (50–57). In spring, there was an increase from mild to moderate stress level (57–68). There was an extreme increase in thermal stress to severe levels in June (82.8 ± 9.5), which persisted through July (89.5 ± 10.7; validated maximum hourly value: 109.8 measured at T = 41.2 °C and RH = 0.78) and August (83.3 ± 7.5), before declining to moderate stress in September. Analysis of the hourly record confirmed that THI exceeded 72 (moderate stress onset) for a daily mean of 14.3 h in July and 11.6 h in August, indicating that thermal relief during cooler nocturnal hours was limited but not absent. The night-time minimum THI (22:00–06:00 window) averaged 78.4 in July and 75.1 in August, remaining within the high-stress zone throughout (76.6 ± 9.1) and mild stress in October (62.1 ± 5.6).

**Table 1:** Monthly outdoor THI (mean ± SD) for 2025 (*n* = 8760 hourly observations).

Month	*n* (h)	Mean	SD	Stress Category	Study Period
January	744	50.84	5.38	No stress (<68)	—
February	672	49.97	3.90	No stress (<68)	—
March	744	56.29	7.44	No-to-mild stress (<68) [indoor THI 62.9 is below the mild-stress threshold]	—
April	720	58.99	7.04	No stress (<68)	—
May	744	67.54	7.49	No stress (<68)	✓
June	720	82.80	9.52	Severe (≥82)	✓
July	744	89.54	10.74	Severe (≥82)	✓
August	744	83.31	7.49	Severe (≥82)	✓
September	720	76.61	9.09	Moderate (72–78)	✓
October	744	62.12	5.57	No stress (<68)	✓
November	720	58.50	5.85	No stress (<68)	—
December	744	51.89	4.44	No stress (<68)	—

SD = within-month hourly variability. July verified maximum = 109.8 (T = 41.2 °C, RH = 0.78). ✓ = study period.

### 3.2. Outdoor Versus Indoor THI: May–October 2025

**Figure 2:**
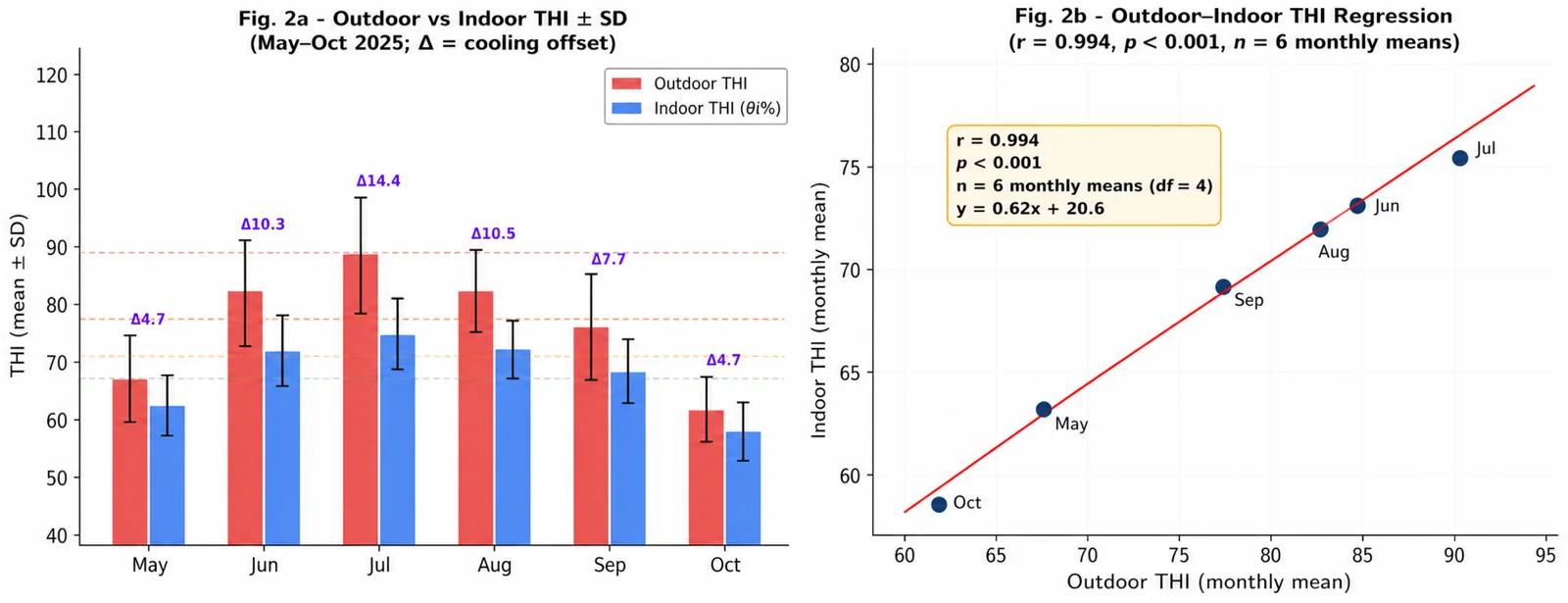
(**a**) Monthly mean outdoor and indoor Temperature–Humidity Index (THI) (mean ± SD) during the study period (May–October 2025). Red bars represent the monthly mean outdoor THI, whereas blue bars represent the monthly mean indoor THI measured inside the farrowing house. Purple Δ labels above each pair of bars indicate the monthly cooling effect (ΔTHI = outdoor THI − indoor THI). Horizontal dotted lines denote the NRC/Thom (1959) THI stress thresholds (mild, moderate, high, and severe heat stress). Error bars indicate ±1 SD. (**b**) Linear regression between monthly mean outdoor and indoor THI values. Blue circles represent the monthly observations (May–October), and the red solid line represents the fitted linear regression (r = 0.994, *p* < 0.001, *n* = 6 monthly means).

The results for indoor–outdoor THI comparison are shown in Table 2 and Figure 2 below. Indoor cooling reduced the outdoor THI, and the monthly reduction (ΔTHI) was from 3.7 in October to 14.3 in July. Despite this attenuation, indoor THI in May (62.9 ± 5.2) was below the mild-stress threshold (<68), indicating no-to-mild stress conditions, and at moderate stress levels (72–78) in June (72.4 ± 6.2), July (75.3 ± 5.9), and August (72.8 ± 4.8), and at mild stress levels (68–72) in September (68.9 ± 5.6). The building offered good but inadequate protection from the extreme heat. The relationship between the average THI values outdoors and indoors over six months of study (df = 4) was very close to being perfectly correlated: r = 0.994, *p* < 0.001, Indoor THI = 0.621 Outdoor + 20.66.

### 3.3. Sow and Litter Characteristics

The descriptive statistics of all enrolled sows are described in Table 3. The total litter size was 15.20 ± 3.85 pigs per sow (ranging from 3 to 24). The effective number of nursed piglets after litter balancing was set to 11.91 ± 1.03 pigs (96.7% weaned, 12 pigs).

### 3.4. Monthly Feed Intake and Farrowing-Batch Performance

**Figure 3:**
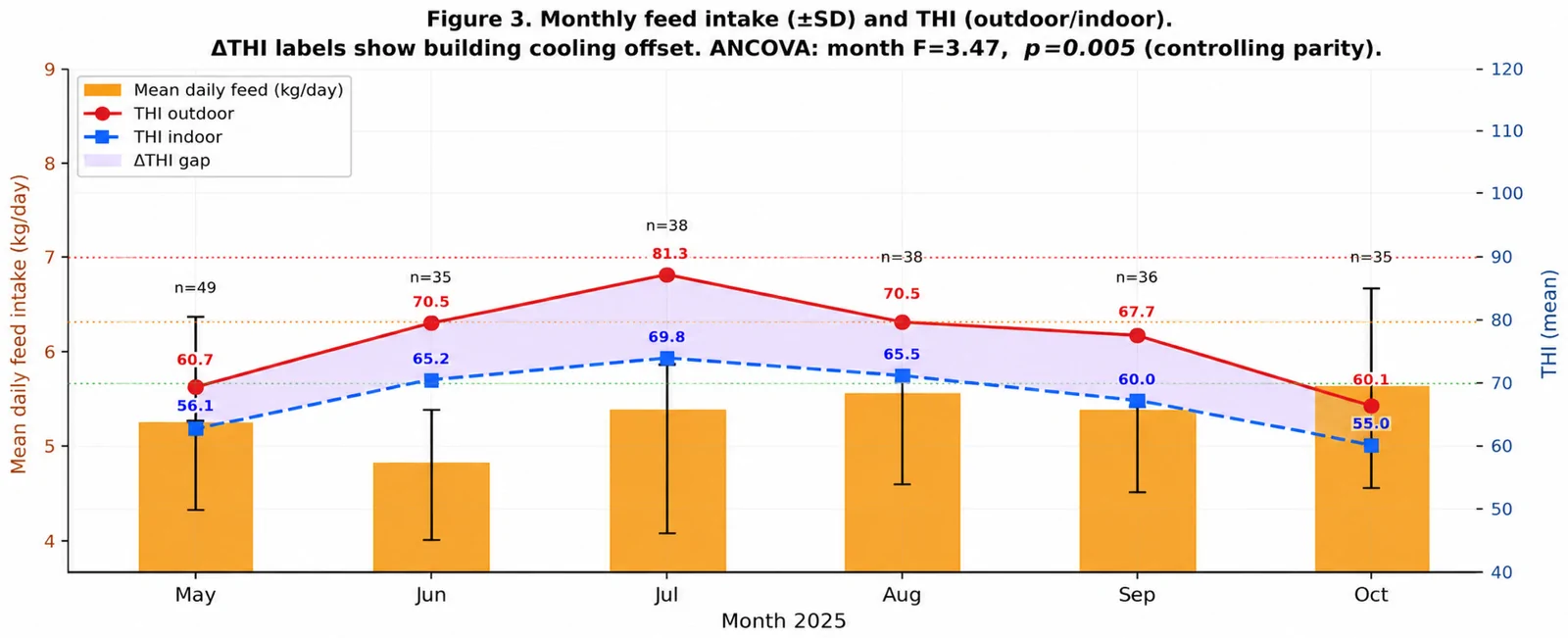
Monthly mean daily feed intake (orange bars, ±SD) in lactating sows by farrowing month (May–October 2025), with red solid line (●) representing the monthly mean outdoor THI (THI_out) and blue dashed line (■) representing the monthly mean indoor THI (THI_in), plotted on the right y-axis. Purple Δ labels indicate the monthly cooling effect (ΔTHI = outdoor THI − indoor THI) achieved by the building. Error bars on the bars indicate ±1 SD of mean daily feed intake. ANCOVA showed a significant effect of month on feed intake after controlling for parity (F = 3.47, *p* = 0.005).

The results of monthly performance are shown in Table 4 and Figure 3. One-way ANOVA indicated that there is a significant month effect on the mean daily feed intake (F = 2.69; df = 5; *p* = 0.022). However, ANCOVA, adjusted for batch parity composition, showed that the month effect was not dependent on batch parity composition (F = 3.00; df = 5; *p* = 0.012), although parity continued to be highly significant (F = 12.99, *p* < 0.001). The significant contrasts between months according to Tukey’s HSD post hoc analysis were found between June vs. August (difference of means = −0.793 kg/day; p_adj = 0.018) and June vs. October (difference of means = −0.815 kg/day; p_adj = 0.014). No other pairwise month contrasts were significant. Accordingly, June is assigned the distinct compact letter display (CLD) superscript b, while May, July, August, September, and October are denoted a (Table 4). The two significant contrasts (June vs. August, p_adj = 0.018; June vs. October, p_adj = 0.014) together define the b grouping for June. Mean parity per batch: May 3.18, June 3.05, July 2.26, August 3.06, September 3.65, October 3.09.

### 3.5. Lactation Feed Intake Curves (Days 1–30)

**Figure 4:**
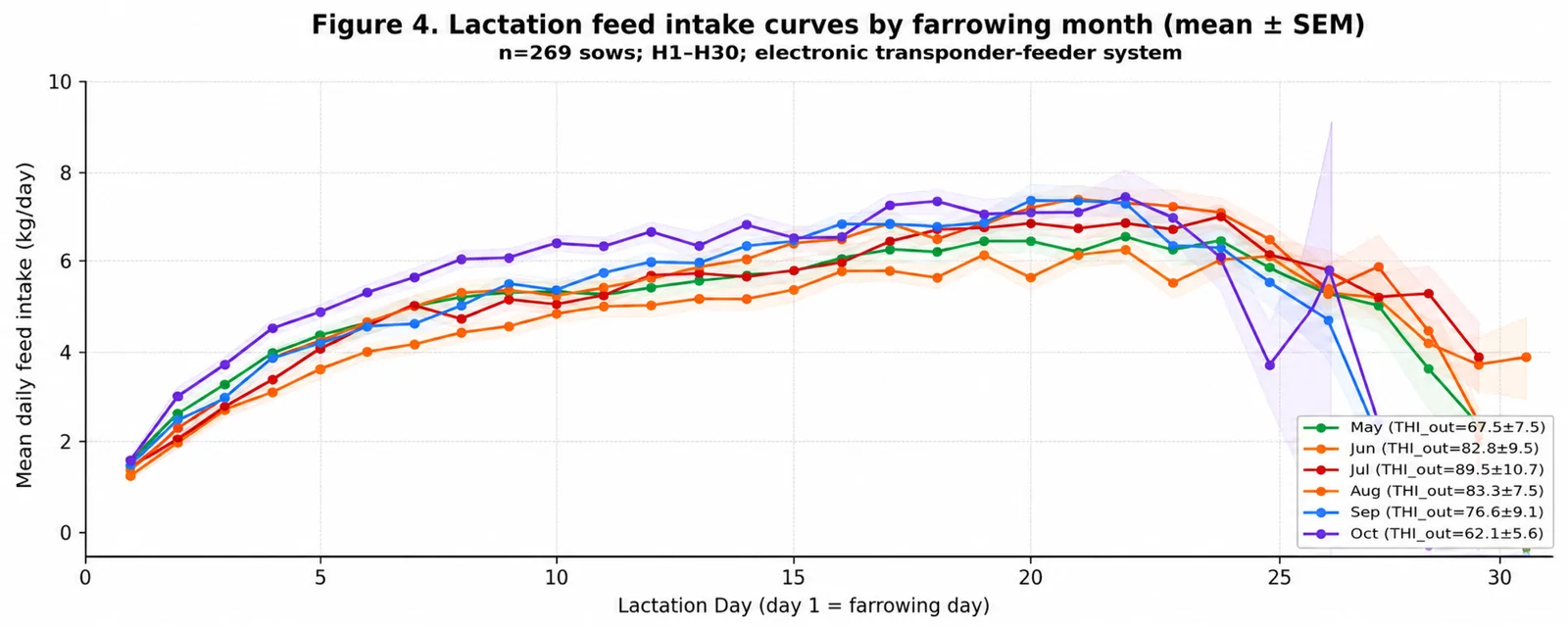
Lactation feed intake curves by farrowing month (mean ± SEM; *n* = 269 sows; days 1–30; GESTAL Solo+ electronic transponder-feeder system; unique ear-tag linkage).

The lactation feed intake trends for individual sows according to their month of farrowing have been illustrated in Figure 4. All farrowing months exhibited the upward sigmoid trend in that starting at about 1.55 kg/day on Day 1, a grand mean peak of 6.89 kg/day was observed on Day 22 before experiencing a pre-weaning reduction to 3.96 kg/day on Day 30.

### 3.6. Effect of Parity on Feed Intake and Litter Performance

**Figure 5:**
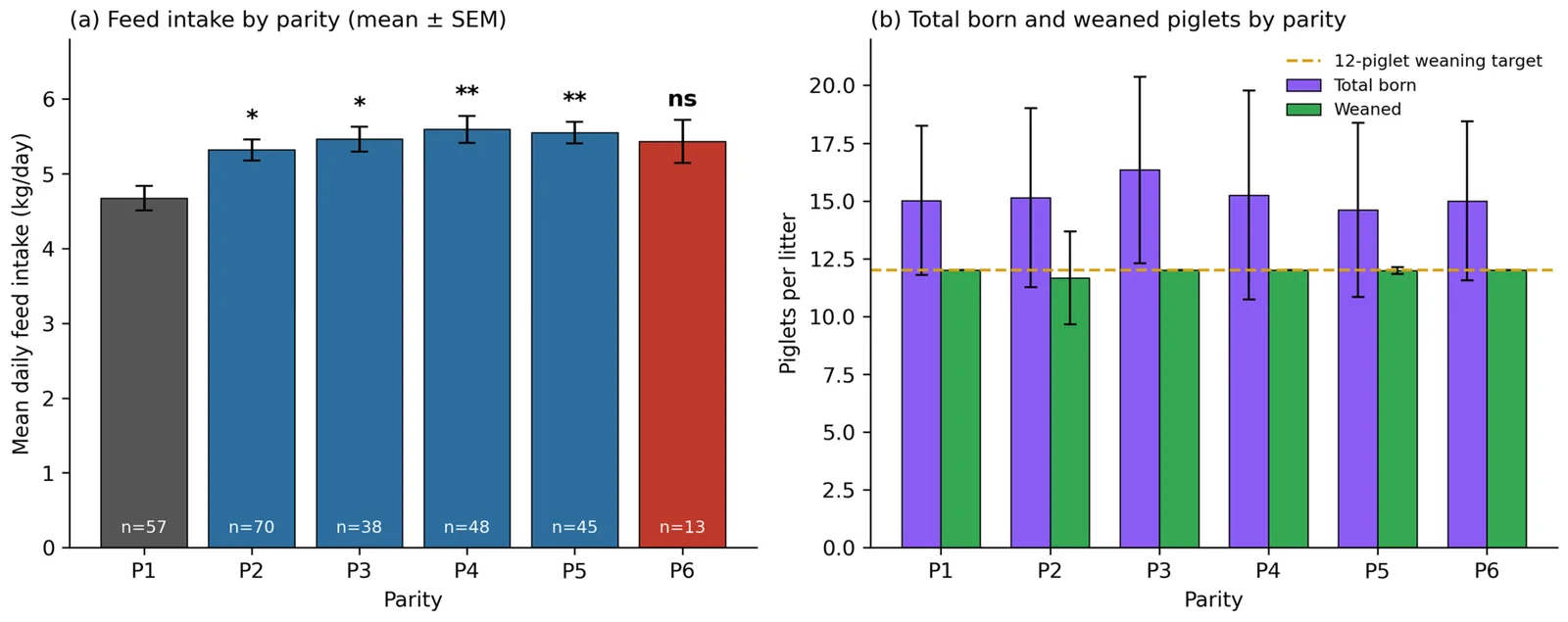
(**a**) Mean daily feed intake (±SEM) by parity, recomputed from the raw sow-level records; significance markers (*, **) vs. P1 from Tukey’s HSD, ns = not significant (P6). Error bars now show the standard error of the mean, consistent with the statistic Tukey’s HSD is based on (see Table 5 for *n*, SD, and SEM per group). (**b**) Total born and weaned piglets by parity (±SD); dashed gold line = 12-piglet weaning target.

Parity turned out to be the most robust independent variable predicting daily average feed intake after performing Bonferroni correction (r = 0.203, p_Bonf = 0.008; ANOVA F = 4.65, *p* < 0.001; Figure 5). Significant differences between groups were established by Tukey’s HSD method between P1–P2 (*p* = 0.007), P1–P3 (*p* = 0.006), P1–P4 (*p* = 0.001), P1–P5 (*p* = 0.002); none of the differences between P2 and other groups were significant. Compact letter display (CLD) superscript values for letters displayed in Table 5: P1 is represented by b (statistically lower than P2–P5), P2–P5 are represented by a, while P6 is represented by ab (not statistically different from both P1 [*p* = 0.239] and P2–P5 [all *p* ≥ 0.897], due to low power for *n* = 13). Feed intake increased from P1 (4.67 ± 1.26 kg/day) to P4 (5.59 ± 1.26 kg/day), then stabilized through P5 (5.55 ± 0.97) and P6 (5.43 ± 1.04).

### 3.7. Litter Balancing: Distribution and Effect on Feed Intake

**Figure 6:**
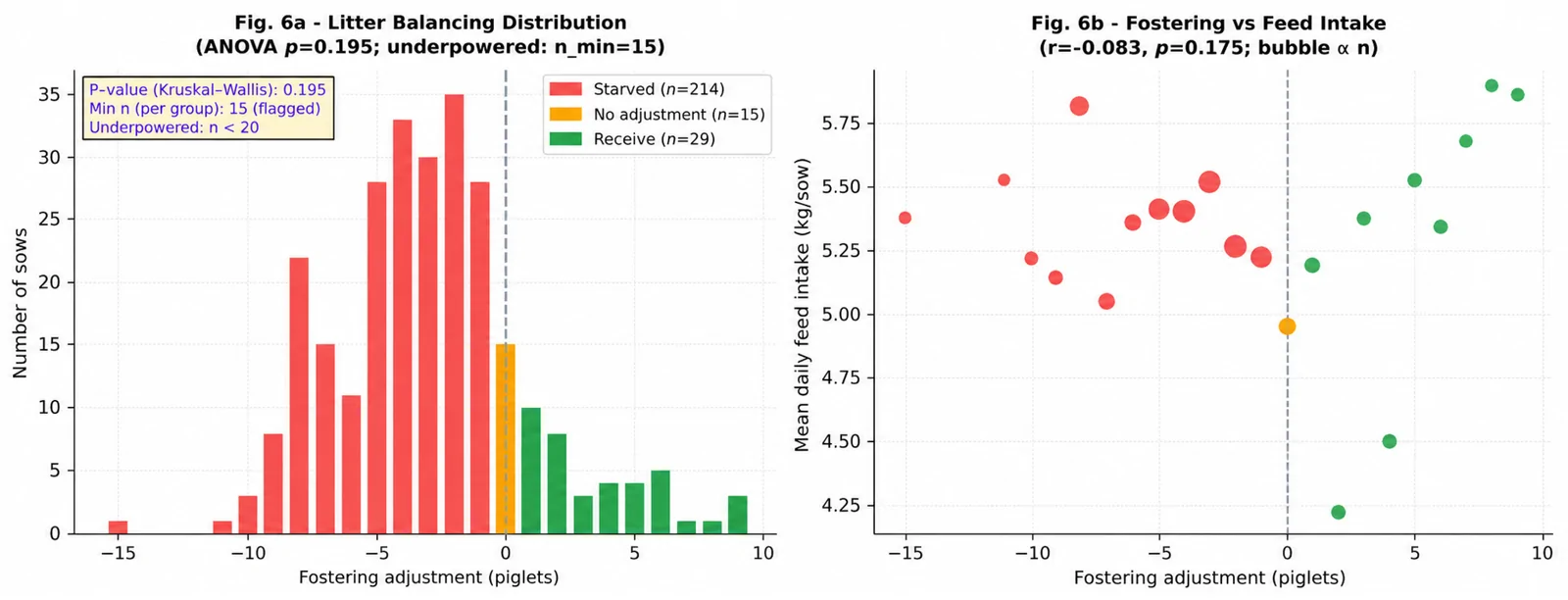
(**a**) Distribution of fostering adjustments (negative = donated, positive = received). (**b**) Mean daily feed by fostering adjustment level (bubble size ∝ *n* sows; r = −0.047, *p* = 0.44). Note: ANOVA *p* = 0.195 with n_min = 15 (minimum detectable difference = 0.51 kg/day for 80% power; observed difference = 0.40 kg/day).

Figure 6 and ANOVA (F = 1.65, *p* = 0.195) show no statistically significant difference in mean daily feed intake across fostering groups. Donors (*n* = 215, 5.35 ± 1.16 kg/day) numerically consumed more than recipients (*n* = 39, 5.06 ± 1.37 kg/day) and unadjusted sows (*n* = 15, 4.95 ± 1.03 kg/day) without statistical significance. A post hoc power analysis reveals that with *n* = 15 in the smallest group, the study had 80% power to detect only differences ≥0.51 kg/day (Cohen’s f = 0.25); the observed maximum difference was 0.40 kg/day. Accordingly, *p* = 0.195 should be interpreted as insufficient statistical power to exclude a clinically meaningful difference rather than confirmed absence of effect. Litter balancing achieved 12 weaned piglets in 269/272 sows (96.7%).

### 3.8. Pearson Correlations with Mean Daily Feed Intake

**Figure 7:**
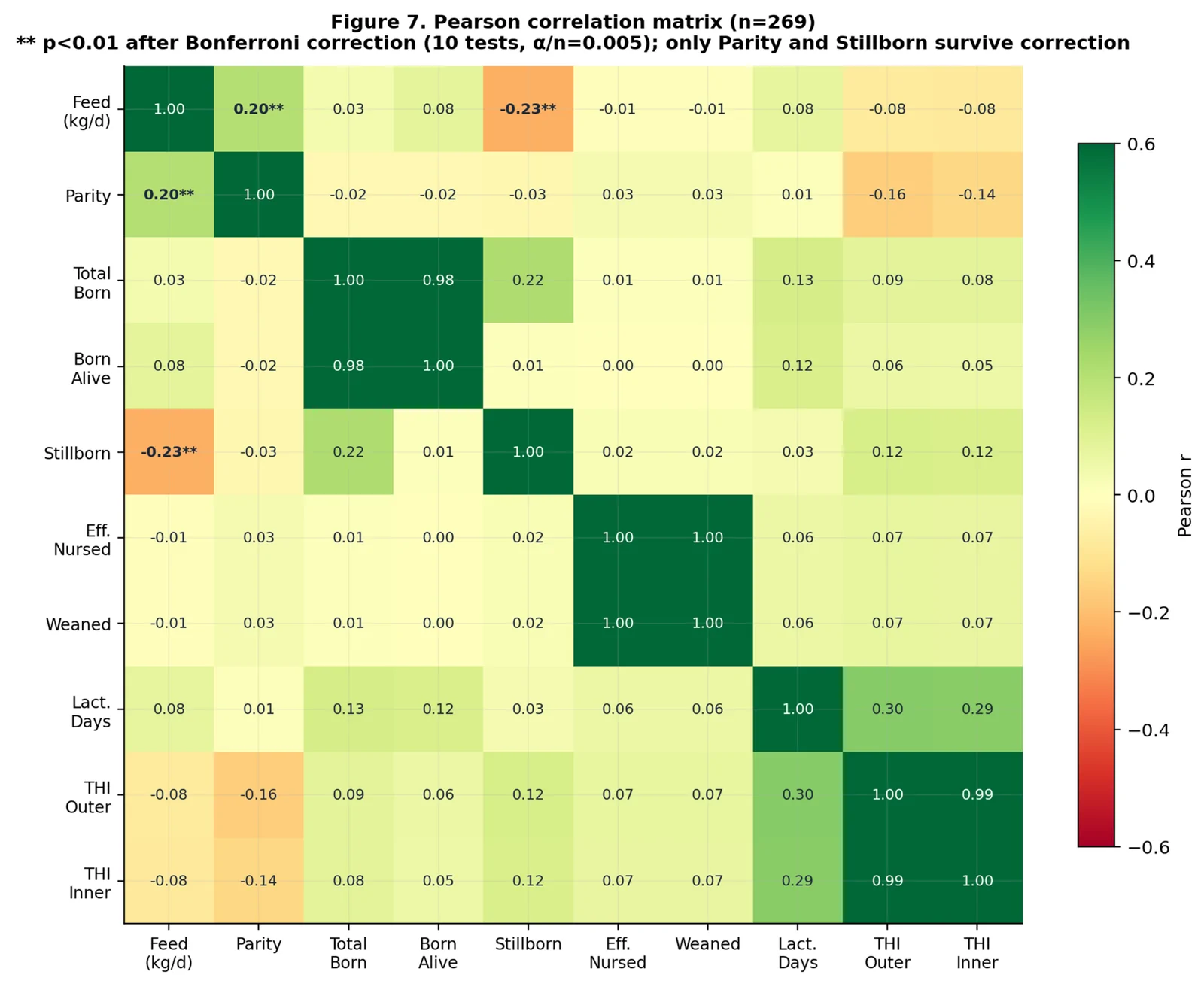
Pearson correlation matrix of sow performance and environmental variables (*n* = 269). ** = significant after Bonferroni correction (10 tests, α/*n* = 0.005); only parity and stillborn survive.

Table 6 presents Pearson correlations with both uncorrected and Bonferroni-corrected *p*-values (10 tests, α/*n* = 0.005), visualized in Figure 7. After correction, only parity (r = 0.203, p_Bonf = 0.008) and stillbirth count (r = −0.221, p_Bonf = 0.002) remained significant. Lactation length (p_raw = 0.007; p_Bonf = 0.070) and effective nursed piglets (p_raw = 0.034; p_Bonf = 0.343) did not survive correction. The uniformly low correlations between THI variables and individual sow feed intake (r = −0.065 to −0.084) are consistent with the batch-level nature of the THI exposure variable: with only 6 unique monthly THI values assigned to 268 sows, the effective degrees of freedom for any THI–outcome association are four (not 267), and individual-level correlations overstate statistical precision (pseudoreplication). This should be interpreted as a design constraint, not as evidence against a biological effect of thermal stress on feed intake. THI correlations (outdoor, indoor, ΔTHI) were non-significant at both individual-sow level (r = −0.065 to −0.084, all *p* > 0.13) and the correct monthly-aggregate level (*n* = 6 monthly means, df = 4: THI_out r = −0.335, *p* = 0.516; THI_in r = −0.352, *p* = 0.494; ΔTHI r = −0.299, *p* = 0.565).

### 3.9. Daily Resolution Feed Intake Model (Sow-Day Level)

**Figure 8:**
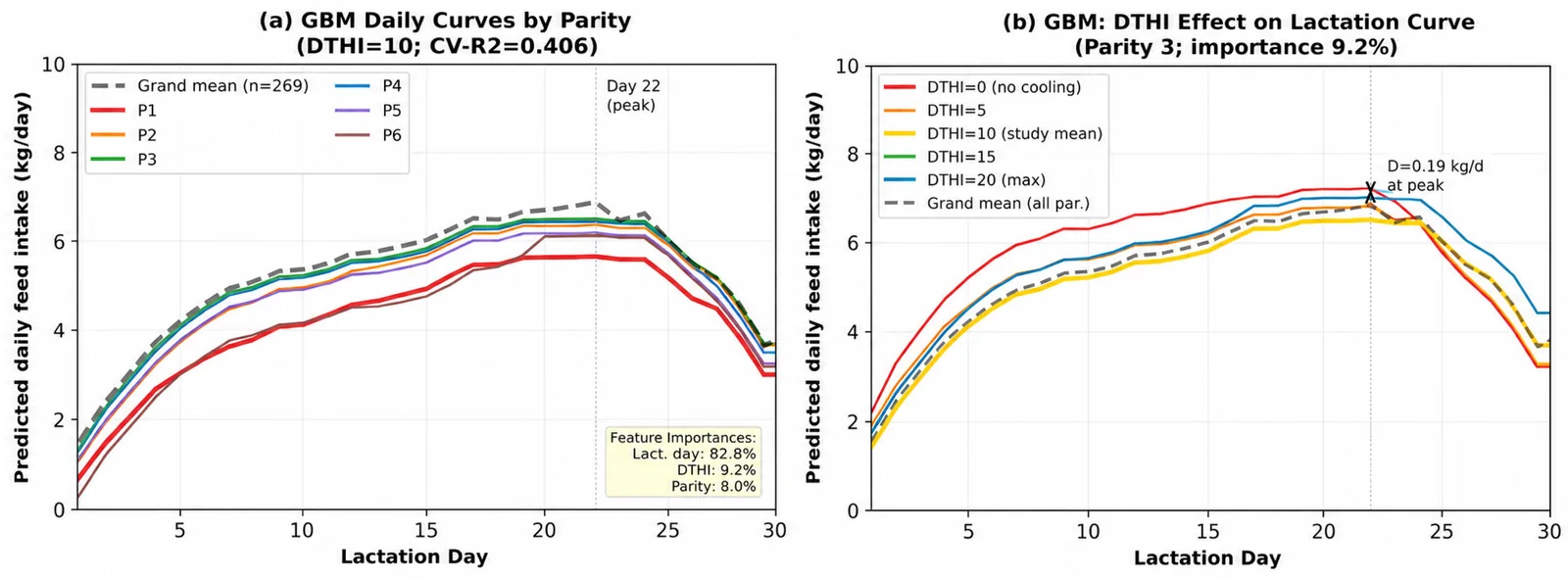
Daily lactation-curve model predictions (Gradient Boosting Regressor; 150 trees, depth = 2; GroupKFold CV-R^2^ = 0.406 ± 0.045; *n* = 6728 sow-days, 268 sows). (**a**) Predicted daily curves by parity (P1–P6) at ΔTHI = 10; dashed = study grand mean (*n* = 269). (**b**) ΔTHI effect on the predicted lactation curve for P3 across 30 lactation days; difference at day 22 between ΔTHI = 0 and ΔTHI = 20 is annotated. Feature importances: lactation day 82.8%; ΔTHI 9.2%; parity 8.0%.

To test whether individual-level feed intake prediction is achievable from the available predictors when the outcome is defined at a finer temporal resolution, and to exploit the substantial within-sow information contained in the daily feed intake records (Figure 4), an alternative formulation was developed that predicts feed intake for each lactation day, rather than the sow-level mean. The dataset was restructured from one row per sow (*n* = 268) to one row per sow-day (*n* = 6728 observations from the same 268 sows, days 1–30), with parity, lactation day, and ΔTHI as predictors. A Gradient Boosting Regressor (150 trees, max depth = 2, learning rate = 0.1, minimum leaf size = 20) was fitted to this restructured dataset. Cross-validation used GroupKFold (5-fold), with all days from a given sow constrained to the same fold, preventing information leakage between the training and test sets that would otherwise arise from the strong within-sow autocorrelation of daily feed intake. This daily lactation-curve model achieved CV-R^2^ = 0.406 ± 0.045 (RMSE = 1.65 kg/day), a substantial and properly validated improvement over the herd-mean model (CV-R^2^ = −0.10). Model predictions are shown in Figure 8. Feature importance analysis indicated that lactation day was the dominant predictor (82.8% of total importance), capturing the characteristic rise-peak-decline shape of the lactation curve (Figure 4), followed by ΔTHI (9.2%) and parity (8.0%). This refinement demonstrates that the principal source of unexplained variance in the original herd-mean model was not absent predictors but an outcome definition (the 30-day average) that discarded the within-sow temporal structure already present in the electronic feeder data. The daily model retains the same exploratory, single-farm scope as the herd-mean model and requires external validation before individual-level deployment; it is presented as a methodological refinement demonstrating that day-specific prediction is achievable with the existing predictor set and has been implemented as an additional module (Supplementary File S2) alongside the original herd-mean predictor. To assess whether this improvement was specific to the Gradient Boosting algorithm or reflected the underlying nonlinear shape of the lactation curve, four alternative model classes—Linear OLS, a Linear Mixed Model with a sow-level random intercept, a Generalized Additive Model with a smooth term on lactation day, and Random Forest—were fitted to the same sow-day dataset under identical sow-grouped 5-fold cross-validation. Both linear approaches (OLS and the Mixed Model) plateaued at CV-R^2^ ≈ 0.23, essentially indistinguishable from one another, while the three nonlinear approaches (GAM, Random Forest, Gradient Boosting) clustered around CV-R^2^ ≈ 0.38–0.39. This comparison indicates that the predictive gain reported above is driven primarily by capturing the nonlinear rise-peak-decline shape of the lactation curve rather than by the specific choice of nonlinear algorithm, and that a random-intercept formulation alone, without addressing this nonlinearity, would not have closed the performance gap. The Generalized Additive Model achieved comparable accuracy to Gradient Boosting (CV-R^2^ = 0.375 vs. 0.391) while remaining fully interpretable through its smooth-curve coefficients and is reported here as a transparent benchmark alongside the Gradient Boosting Regressor.

## 4. Discussion

### 4.1. Thermal Environment and Building Cooling Performance

The full-year outdoor THI profile (mean ± SD) confirms the severe and prolonged thermal challenge of Mediterranean pig farming. Outdoor THI reached severe levels (≥82) in June, July, and August (peaking at 89.5 ± 10.7 in July; verified hourly maximum = 109.8) and high levels (78–82) in May, before declining through moderate and mild stress by autumn. 

The adoption of ΔTHI as a more operational metric to make decisions on farm, as opposed to the THI values considered separately, can be viewed in the following manner. ΔTHI gives us a direct measure of the cooling efficiency of our buildings, which is the actual variable we can control, thus making it possible to compare performances across different farms in diverse climatic zones. A farm with ΔTHI = 14 will certainly give greater protection against heat stress to the sows compared to an environment with ΔTHI = 4. This means that a ΔTHI of 14 is significantly better than a ΔTHI of 4. Huynh et al. (2005) [17] also found that at temperatures above 25 °C, the humidity level is an important factor in increasing the thermal load on pigs [17]. The result obtained, showing that the indoor–outdoor regression coefficient was 0.621 (Indoor THI = 0.621 × Outdoor + 20.6; r = 0.994), indicates that although the structure dampens the outdoor variability, it does not eliminate it; the value of 20.6 obtained shows that there is still some elevation due to heat production by animals and the insulative effect. The results obtained above are similar to findings from Mediterranean swine houses as documented by Huynh et al. (2006) [18], who found that temperature differences between indoor and outdoor were 3–8 degrees Celsius in naturally ventilated structures under similar conditions [18]. It should, however, be noted that greater dampening was achieved through forced ventilation under peak periods of stress. Godyń et al. [19] further showed that measuring peripheral and core body temperatures in swine depends on validating environmental limits. It should be noted that the monthly mean THI values used throughout represent a temporal average that smooths the diurnal cycle; statements about ‘severe and prolonged’ heat stress refer primarily to daytime peak conditions. An alternative and complementary metric—the Thermal Loading Index (TLI = indoor THI − 68, set to 0 when indoor THI < 68)—was computed for each study month as suggested by reviewers, to quantify the absolute thermal burden above the no-stress threshold experienced by the sows: TLI ranged from 0 (May, October) to 7.3 (July), providing a sow-impact perspective that complements the building-efficiency perspective offered by ΔTHI. [19].

### 4.2. Parity and Stillbirth as Primary Predictors of Feed Intake

According to Mullan & Williams (1989) [20], primiparous (P1) sows start lactation with poorer body condition than the multiparous (P2+) ones and are required not only to nurse the litter but also to gain weight themselves [20]. Thus, it is natural for the first category to consume less food than the second one. Kim et al. (2009) [21] found that the amino acid ratio should be higher in the diet of primiparous sows than in multiparous ones since the physiological needs of both groups are different [21]. According to Prunier et al. (1997) [5], the impact of heat on this issue cannot be neglected [5]. In this research, it was discovered that primiparous sows were characterized by a particularly significant feed intake decline when exposed to elevated temperatures. Although the current study could not demonstrate a significant parity by month interaction due to limited power, the trend of the data supports the confounding impact of the parity composition on the differences in feed consumption found between the months (June P1 average parity = 3.05; July P1 group = 2.26). In practical terms, the effect of primiparity confirmed via Tukey’s HSD testing (effect size Cohen’s d = 0.72, medium to large effect) can justify the separation of feeding programs for P1 animals, especially in high THI months when both factors act cumulatively, resulting in the greatest nutritional disadvantage [22].

The statistically significant inverse relationship between the number of stillbirths and feed intake (r = −0.221, p_Bonf = 0.002) holds even following Bonferroni correction and constitutes an interesting biological finding. It is, however, impossible to determine causality given the nature of the data, which suggests that antepartum hypophagia results in reduced uterine contractility, making farrowings more difficult, or vice versa, that farrowings themselves result in poor periparturient appetite. Indeed, the link between pre- and post-farrowing nutrition and performance during farrowing and lactation has been studied before. As reported by Quiniou and Noblet (1999) [2], sows with the greatest depression in feed intake at the end of gestation because of heat stress had the smallest weaning weights and largest losses of piglets, indicating a cause-and-effect relationship. Similarly, Renaudeau and Noblet (2001) [16] observed that high ambient temperature in late gestation and early lactation impaired colostrum production, which could independently affect neonatal viability [16]. The present association should not be interpreted causally without prospective timed feed intake data before and during farrowing, and ideally after adjusting for parity, litter size, gestation length, and farrowing duration as covariates. The finding is presented as hypothesis-generating and warrants a dedicated prospective study with individual farrowing event monitoring.

### 4.3. Month Effect and Parity Confounding

These findings reinforce the importance of controlling for parity composition when comparing farrowing-batch performance across seasonal cohorts, a point that is frequently overlooked in farm-level benchmarking. The absence of a direct individual-level THI–feed intake association in the current dataset is not unexpected given the statistical limitations of assigning a batch-level variable (six unique monthly THI values) to 269 individual sows. Renaudeau et al. (2011) [8], in a meta-analysis of 17 studies on growing pigs, reported a curvilinear relationship between ambient temperature and feed intake, with meaningful individual-level effects observable only when within-study temperature variation exceeded approximately 5 °C above the thermoneutral zone [8]. The six-batch design of the present study, while commercially pragmatic, provides df = 4 for THI effects. A formal two-way ANOVA with a parity × month interaction term was also fitted to the current dataset (F = 1.24, *p* = 0.247); the interaction was non-significant, consistent with limited power given the unbalanced design (many parity × month cells had *n* < 5). The non-significant interaction does not rule out true confounding of the month effect by parity composition; ANCOVA with mean batch parity as a covariate (Section 2.7 and Section 3.4) is the preferred approach to address this confound in the current design at the appropriate aggregate level—insufficient to detect small associations. Future studies with greater temporal resolution (e.g., weekly or fortnightly farrowing batches across the same season) would substantially increase statistical power for detecting individual-level THI effects. However, Black et al. (1993) [4] had shown that the temperature threshold for hypophagia in lactating sows was about 20–22 °C [4]. This was substantially lower than the levels of indoor THI obtained in this study from June–September, which suggests high validity of any THI–feed intake relationship, despite lack of statistical significance.

### 4.4. Litter Balancing: Power Considerations

The litter balancing ANOVA (F = 1.65, *p* = 0.195) should be interpreted considering the post hoc power analysis. With *n* = 15 in the no-adjustment group, the study had 80% power to detect only differences ≥ 0.51 kg/day (Cohen’s f = 0.25); the observed maximum group difference was 0.40 kg/day, below this threshold. The result therefore represents insufficient statistical power rather than confirmed nutritional neutrality. A prospectively powered study with *n* ≥ 157 per fostering category would be required to definitively characterize the relationship between litter balancing and individual sow feed intake.

The litter balancing protocol employed in this study, which targeted 12 weaned piglets per sow via cross-fostering, achieved the target in 96.7% of cases and standardized effectively nursed piglets to 11.91 ± 1.03. This near-universal standardization explains the non-significance of effectively nursed piglets as a predictor of mean daily feed intake in the regression model (*p* = 0.814): when there is almost no between-sow variation in litter size, the variable carries minimal predictive information. This is a methodological strength of the litter balancing protocol from a welfare and production perspective, but it constrains the ability to study litter-size effects on sow feed intake within the balanced cohort. Earlier work by Mullan and Williams (1989) [20] and Noblet and Etienne (1987) [1] was conducted in cohorts without systematic litter balancing, making direct comparisons with the present study difficult; their larger between-sow variation in litter size allowed litter-size effects on milk production and feed intake to be characterized. Future observational studies of sow nutrition that maintain natural litter size variation would provide complementary data. The underpowered fostering ANOVA (*p* = 0.195, n_min = 15) further illustrates the practical challenge of studying three-category comparisons in production settings where the ‘no-adjustment’ group is necessarily small.

### 4.5. The Daily Resolution Feed Intake Model in the Context of Precision Livestock Farming

Precision Livestock Farming (PLF) represents a relatively new way of approaching the production process by means of real-time monitoring and automated analysis for the optimization of individual animals’ well-being, performance, and resource use in commercial livestock enterprises [9,10,23]. Indeed, PLF can be seen as a move away from batch-based periodic assessments, which used to be the standard practice in the pig production sector, and into using sensor data collected either at the animal or the herd level and analyzed through models to make real-time predictions and recommendations [9]. The combination of data on individual electronic feeding stations (like the GESTAL Solo+ system employed in this project) with information from environmental monitoring is one of the most obvious applications of PLF: according to Pomar et al. (2009) [11], precision feeding of individual growing–finishing pigs based on appetite and body weight data led to up to 5–10% savings in the cost of feed use and up to 20–25% reduction in nitrogen emissions compared to group-based feeding programs. According to Neethirajan (2020) [24], new sensors can be applied to swine PLF, and thermal stress monitoring is considered one of the priority areas for their implementation due to the large amounts of monetary losses incurred from heat stress [24]. St-Pierre et al. (2003) [25] estimated it to be approximately USD 2.4 billion per year for US livestock sectors, including swine PLF (USD 299–316 million) [25]. In addition, according to Papatsiros et al. (2026) [12], the Mediterranean climate becomes increasingly hot every year, adding even more weight to the need for affordable monitoring systems for detecting thermal changes [12].

The predictive ability of the model (R^2^ = 0.049; CV-R^2^ = −0.10) affirms its classification as exploratory: it does not provide better prediction of feed consumption by each sow than the grand mean of the herd. This model will be useful only on the herd scale, i.e., for thermal referencing and herd stratification based on parity. This model was tested in one farm setting only, and thus, needs further validation in other farms under various conditions. The ΔTHI regression parameter was not significantly different from zero at the individual sow level (β = −0.017, *p* = 0.399). The tool nonetheless provides a framework for quantifying and comparing building cooling performance across farms and seasons via the ΔTHI metric. The current model cannot yet be used for prediction at the individual sow level and should be considered only as an exploratory framework requiring external validation. The present study provides the initial framework for developing a THI-based feed intake prediction system that requires further validation before commercial implementation.

Within these acknowledged constraints, the proof-of-concept framework offers four practical contributions at the herd level. First, the ΔTHI metric provides a farm-comparable, quantitative benchmark for building cooling performance; farms achieving ΔTHI ≥ 10 in summer months demonstrate superior thermal buffering relative to those with ΔTHI < 5. Second, the Tukey’s HSD-confirmed primiparity deficit (Cohen’s d = 0.72, P1 vs. P2+) provides statistical justification for differentiated first-parity feeding programs. Third, when individual ear-tag records are available, comparison of a sow’s intake against the farrowing-batch mean (not the model prediction, given CV-R^2^ = −0.10) provides a practical screening signal. Fourth, the cross-platform browser implementation makes thermal benchmarking accessible without software installation. Fifth, the daily lactation curve refinement (Section 3.9) demonstrates that day-specific feed intake prediction is achievable from the same predictor set (parity, ΔTHI, lactation day) once the outcome is redefined at the sow-day level rather than the sow-mean level, raising CV-R^2^ from −0.10 to 0.406 ± 0.045 under properly grouped cross-validation—a methodological illustration of how outcome granularity, rather than predictor availability alone, constrained the original model’s performance.

Priority variables for future model iterations include: (a) individual sow live weight and body condition score (BCS) at farrowing, which Mullan and Williams [20] demonstrated to be among the strongest individual-level predictors of lactation feed intake and body reserve mobilization; (b) individual daily water intake, which mediates part of the HS–hypophagia relationship via evaporative cooling of the gastrointestinal tract and was not measured by the GESTAL Solo+ system in this study; and (c) continuous daily-level THI matched to each sow’s specific lactation days, which would resolve the pseudoreplication inherent in batch-level THI assignment. Nutrient conversion efficiency and milk production data were also unavailable in this study. Future model iterations should incorporate individual sow body condition score and live weight at farrowing, which Mullan and Williams (1989) [20] demonstrated to be among the strongest individual-level predictors of lactation feed intake and body reserve mobilization. Continuous daily-level THI matched to each sow’s specific lactation days would resolve the pseudoreplication inherent in batch-level THI assignment and would substantially increase statistical power for detecting individual thermal effects. Water intake, which mediates part of the HS–hypophagia relationship via evaporative cooling of the gastrointestinal tract, and time-of-day feeding patterns were unavailable in this dataset but would substantially enrich the predictor set [4,17]. Machine learning approaches—such as random forest, gradient-boosted regression trees, or long short-term memory networks applied to time-series data from electronic feeders—could be explored if multi-farm datasets with richer individual-level predictors become available, provided that cross-validated performance is reported and validation across farms, genetics, and climates is completed before any individual-level application is claimed [24]. The sow-day Gradient Boosting model presented in Section 3.9 (CV-R^2^ = 0.406, *n* = 268 sows on one commercial farm in the Mediterranean region) already demonstrates that individual daily feed intake can be predicted with genuine, properly cross-validated accuracy from a small, readily available predictor set (parity, ΔTHI, lactation day); future work using additional predictors such as body condition score, live weight, and water intake would be expected to improve on this further. As a methodological sensitivity check, the Gradient Boosting refinement was benchmarked against a Linear Mixed Model and a Generalized Additive Model fitted to the same sow-day data under identical cross-validation. The Linear Mixed Model, despite including a sow-level random intercept, performed no better than pooled linear regression for out-of-sample prediction of new sows (CV-R^2^ = 0.231 vs. 0.230), confirming that random-effects structure alone does not address the nonlinearity of the lactation curve. The Generalized Additive Model, by contrast, achieved performance close to that of Gradient Boosting (CV-R^2^ = 0.375 vs. 0.391) while offering directly interpretable smooth-curve coefficients and represents a promising alternative for future implementations of this daily resolution feed intake model.

Future research should validate the daily resolution feed intake model across multiple commercial farms representing different climatic regions, housing systems, and genetic lines. Integration of additional physiological and behavioral indicators, including body temperature, respiration rate, activity monitoring, thermal imaging, body weight, and milk production, is expected to improve predictive performance substantially. The incorporation of machine-learning algorithms, Internet of Things (IoT) technologies, wearable sensors, and Digital Twin platforms could facilitate real-time prediction of heat stress responses and enable adaptive environmental control, precision feeding, and early-warning systems. Such integrated approaches have considerable potential to improve animal welfare, production efficiency, and the resilience of pig production systems under future climate change scenarios.

## 5. Conclusions

This study demonstrates severe and prolonged outdoor heat stress in Mediterranean summer (THI up to 89.5) that mechanical ventilation attenuated but did not eliminate indoors (ΔTHI 3.7–14.3). Parity and stillbirth count were the only Bonferroni-significant correlates of individual sow feed intake; restructuring feed intake at the sow-day level, with proper grouped cross-validation, enabled genuinely predictive modeling (CV-R^2^ = 0.406). External validation across farms, genetics, and climates is required before any individual-level or commercial application.

## Figures and Tables

**Table 2:** Monthly indoor and outdoor THI comparison (May–October 2025; mean ± SD). Outdoor–indoor r = 0.994, *n* = 6 monthly means (df = 4), *p* < 0.001.

Month	THI_out (Mean ± SD)	THI_in (Mean ± SD)	ΔTHI	*n* Out (h)	*n* In (h)	Indoor Stress	TLI (Units)
May	67.54 ± 7.49	62.87 ± 5.21	4.67	744	738	No stress (<68)	0.00 (no burden)
June	82.80 ± 9.52	72.35 ± 6.18	10.45	720	718	Moderate	4.35
July *	89.54 ± 10.74	75.25 ± 5.93	14.29	744	742	Moderate (outdoor: Severe)	7.25
August	83.31 ± 7.49	72.81 ± 4.82	10.50	744	740	Moderate	4.81
September	76.61 ± 9.09	68.90 ± 5.64	7.71	720	716	Mild	0.90
October	62.12 ± 5.57	58.46 ± 5.02	3.66	744	737	No stress (<68)	0.00 (no burden)

* July: outdoor reached severe stress (≥82) while building cooling reduced indoor to moderate stress (75.3). ΔTHI = outdoor THI_mean − indoor THI_mean. Outdoor–indoor r = 0.994, *n* = 6 monthly means (df = 4), *p* < 0.001. Indoor hours differ due to 209 records excluded in calibration review.

**Table 3:** Overall descriptive statistics for enrolled sows (*n* = 272; feed analyses *n* = 269).

Variable	*n*	Mean	SD	Min	Max	Median
Total born (piglets)	272	15.20	±3.85	3	24	15
Born alive (piglets)	272	14.91	±3.75	3	23	15
Stillborn (piglets)	272	0.24	±0.77	0	5	0
Fostering adjustment (piglets)	272	−3.00	±3.87	−15	+9	−3
Effectively nursed (after balancing)	269	11.91	±1.03	8	23	12
Weaned (piglets)	269	11.91	±1.03	0	12	12
Lactation length (days)	269	24.81	±3.93	3	30 ^†^	25
Parity (P1–P6 in analyses)	271	2.98	±1.55	1	7	3
Mean daily feed (kg/day)	269	5.29	±1.19	0.90	7.65	5.40
Total feed (kg)	269	133.03	±37.80	2.1	210.5	134.75

^†^ Capped at 30 days of recording. Five sows with lact_length 31–47 days: mean_feed reflects days 1–30 only. LW breed (*n* = 9): 5.065 ± 1.064 kg/day vs. F1 (*n* = 263): 5.294 ± 1.196 kg/day; t = 0.567, *p* = 0.571 ns. Nursed effectively equals born effectively + net fostering adjustment (positive means received, negative means donated); the upper limit of 23 represents exceptional cases whereby sows adopted additional artificial rearing following excessive litter mortality in neighboring farrowing crates. The target of 12 weaned piglets was realized in 269/272 sows (96.7%); three sows whose lactation period equaled zero due to peripartum losses are not considered in feed intake analyses (*n* = 269) but included in litter statistics (*n* = 272). A sensitivity analysis that excluded three sows with the lowest lactation duration in the analytical sample group (lact_len = 13 d, *n* = 3) showed little impact on regression coefficients (all within 2%).

**Table 4:** Monthly farrowing-batch performance and climatic data (May–October 2025; mean ± SD). Superscript letters in the feed column: Tukey’s HSD compact letter display (CLD, FWER = 0.05)—means sharing the same letter are not significantly different.

Month	*n*	Feed (kg/d)	Total Born	Weaned	Lact. (d)	Parity †	THI_out	THI_in	ΔTHI	TLI (Units)
May	49	5.282 ± 1.133 ^a^	15.5 ± 3.8	11.7 ± 1.7	24.0 ± 4.2	3.18	67.54 ± 7.49	62.87 ± 5.21	4.67	0.00 (no burden)
June	55	4.810 ± 0.985 ^b^	14.8 ± 4.5	12.0 ± 0.0	25.3 ± 4.6	3.05	82.80 ± 9.52	72.35 ± 6.18	10.45	4.35
July	58	5.335 ± 1.398 ^a^	16.2 ± 3.6	12.0 ± 0.0	26.3 ± 2.4	2.26	89.54 ± 10.74	75.25 ± 5.93	14.29	7.25
August	35	5.603 ± 1.101 ^a^	14.2 ± 3.5	11.7 ± 2.0	25.1 ± 4.9	3.06	83.31 ± 7.49	72.81 ± 4.82	10.50	4.81
September	37	5.456 ± 1.029 ^a^	15.8 ± 4.0	12.0 ± 0.0	23.8 ± 4.2	3.65	76.61 ± 9.09	68.90 ± 5.64	7.71	0.90
October	35	5.626 ± 1.046 ^a^	14.0 ± 3.0	12.0 ± 0.0	23.5 ± 1.6	3.09	62.12 ± 5.57	58.46 ± 5.02	3.66	0.00 (no burden)

Superscript letters (a, b) in feed column: Tukey’s HSD CLD (FWER = 0.05). June (b) significantly lower than August (a) (p_adj = 0.018) and October (a) (p_adj = 0.014); all other pairwise contrasts non-significant. ANOVA: F = 2.69, *p* = 0.022. ANCOVA (|parity): F = 3.00, *p* = 0.012. CLD letters are shown only for feed because it was the sole variable subjected to a formal between-month significance test (ANOVA/Tukey’s HSD); the remaining columns are descriptive monthly summaries (mean ± SD) and were not tested for between-month differences. † Mean parity per batch. All numeric columns: mean ± SD.

**Table 5:** Feed intake and litter performance by parity group (P1–P6; P7, *n* = 1, excluded). Superscript letters in the feed column: Tukey’s HSD CLD (FWER = 0.05)—means sharing the same letter are not significantly different.

Parity	*n*	Feed (kg/d)	Feed SD (SEM)	Total Born	Weaned	Tukey’s HSD vs. P1
P1	57	4.674 ^b^	1.255 (SEM 0.166)	15.0 ± 3.2	12.0 ± 0.0	—(reference)
P2	70	5.318 ^a^	1.157 (SEM 0.138)	15.1 ± 3.9	11.7 ± 2.0	p_adj = 0.007 * (diff = +0.644)
P3	38	5.463 ^a^	1.026 (SEM 0.166)	16.3 ± 4.0	12.0 ± 0.0	p_adj = 0.006 * (diff = +0.789)
P4	48	5.594 ^a^	1.255 (SEM 0.181)	15.2 ± 4.5	12.0 ± 0.0	p_adj = 0.001 ** (diff = +0.920)
P5	45	5.551 ^a^	0.969 (SEM 0.144)	14.6 ± 3.8	12.0 ± 0.1	p_adj = 0.002 ** (diff = +0.877)
P6	13	5.430 ^ab^	1.038 (SEM 0.288)	15.0 ± 3.4	12.0 ± 0.0	p_adj = 0.239 ns (*n* = 13; low power)

Superscript letters (a, b, ab): Tukey’s HSD CLD (FWER = 0.05). P1 b differs from P2–P5 a (all *p* ≤ 0.007); P6 ab does not significantly differ from P1 b (*p* = 0.239) or from P2–P5 a (all *p* ≥ 0.897); no P2–P6 contrasts significant (all p_adj ≥ 0.897). * *p* < 0.05; ** *p* < 0.01. Although P6’s raw mean (5.430) is numerically higher than P2’s (5.318), P6–P1 is non-significant while P2–P1 is significant because P6 has a much smaller sample (*n* = 13 vs. *n* = 70) and therefore a much larger SEM (0.288 vs. 0.138); Tukey’s HSD compares means using the standard error of each mean (which depends on both SD and *n*), not the raw SD or the point estimate alone, so a numerically larger difference can still be statistically weaker when it is estimated with much less precision.

**Table 6:** Pearson correlations with mean daily feed intake. Both raw and Bonferroni-corrected (10 tests, α/*n* = 0.005) *p*-values reported.

Variable	r	*p* Raw	*p* Bonf.	Sig. (Bonf.)	Note
Parity	0.203	0.00076	0.00759	**	Significant after correction
Stillborn	−0.221	0.00024	0.00243	**	Significant after correction
Lact. length (d)	0.164	0.00697	0.06971	ns †	Significant raw; fails correction
Effective nursed	0.129	0.03430	0.34300	ns	Fails correction
Born alive	0.084	0.16763	1.00000	ns	—
THI outdoor ‡	−0.077	0.20764	1.00000	ns	Monthly (*n* = 6): r = −0.335, *p* = 0.516
THI indoor ‡	−0.084	0.17143	1.00000	ns	Monthly (*n* = 6): r = −0.352, *p* = 0.494
ΔTHI ‡	−0.065	0.28715	1.00000	ns	Monthly (*n* = 6): r = −0.299, *p* = 0.565
Total born	0.033	0.58655	1.00000	ns	—
Fostering adj.	−0.083	0.17501	1.00000	ns	Underpowered (n_min = 15)

** *p* < 0.01 after Bonferroni correction. ns = not significant (*p* > 0.05). † p_raw significant but fails Bonferroni correction. ‡ THI variables assigned at monthly batch level (6 unique values); individual-level *n* inflates apparent precision. Pseudoreplication: THI rows have effective df = 4 (6 monthly means), not df = 266; individual-level *n* = 268 artificially inflates apparent precision. At the correct monthly-aggregate level (*n* = 6, df = 4), all THI correlations remain non-significant (all *p* > 0.37). The low r values reflect this design constraint, not the absence of a biological effect of heat stress on feed intake.

## Data Availability

The original contributions presented in this study are included in the article. Further inquiries can be directed to the corresponding author.

## Supplementary Materials

The following supporting information can be downloaded at: https://www.mdpi.com/article/10.3390/life16081306/s1, Supplementary File S1: schematic diagram of the farrowing house; Supplementary File S2: Photos from the trial farm; Supplementary File S3: Equipment for THI monitoring.

## Abbreviations

The following abbreviations are used in this manuscript:

ANOVA	Analysis of Variance
ANCOVA	Analysis of Covariance
BCS	Body Condition Score
BP	Breusch–Pagan
CI	Confidence Interval
CIGR	International Commission of Agricultural and Biosystems Engineering (Commission Internationale du Génie Rural)
CLD	Compact Letter Display
CV	Cross-Validation
CV-R^2^	Cross-Validated Coefficient of Determination
df	Degrees of Freedom
ΔTHI	Difference between Outdoor and Indoor Temperature–Humidity Index (THI_outdoor − THI_indoor)
EMA	European Medicines Agency
ERA5-Land	Fifth Generation ECMWF Atmospheric Reanalysis of the Global Climate—Land Component
GBM	Gradient Boosting Regressor
FASS	Federation of Animal Science Societies
FWER	Family-Wise Error Rate
HSD	Honestly Significant Difference
HS	Heat Stress
HTML	HyperText Markup Language
IPCC	Intergovernmental Panel on Climate Change
JS	JavaScript
KFold	K-fold Cross-Validation
LW	Large White
NRC	National Research Council
OLS	Ordinary Least Squares
P1–P7	Parity 1–7
p_adj	Adjusted *p*-value
p_Bonf	Bonferroni-corrected *p*-value
PLF	Precision Livestock Farming
RH	Relative Humidity
RFID	Radio Frequency Identification
RMSE	Root Mean Square Error
R^2^	Coefficient of Determination
SD	Standard Deviation
SE	Standard Error
SEM	Standard Error of the Mean
THI	Temperature–Humidity Index
THI_in	Indoor Temperature–Humidity Index
THI_out	Outdoor Temperature–Humidity Index
VPD	Vapor Pressure Deficit
VIF	Variance Inflation Factor
TLI	Thermal Loading Index
