# Supplementary material for: Heat Stress, Indoor Microclimate, and Lactation Feed Intake in Commercial Mediterranean Sows: Parity Effects and a Daily Resolution Predictive Model
Source: Life (Basel). 2026 Aug 9;16(8):1306. doi: 10.3390/life16081306 (PMC13514657; doi:10.3390/life16081306)

# Outdoor and Indoor THI Monitoring

## Kestrel 5400 Heat Stress Tracker dataloggers (Nielsen-Kellerman, Boothwyn, PA, USA)

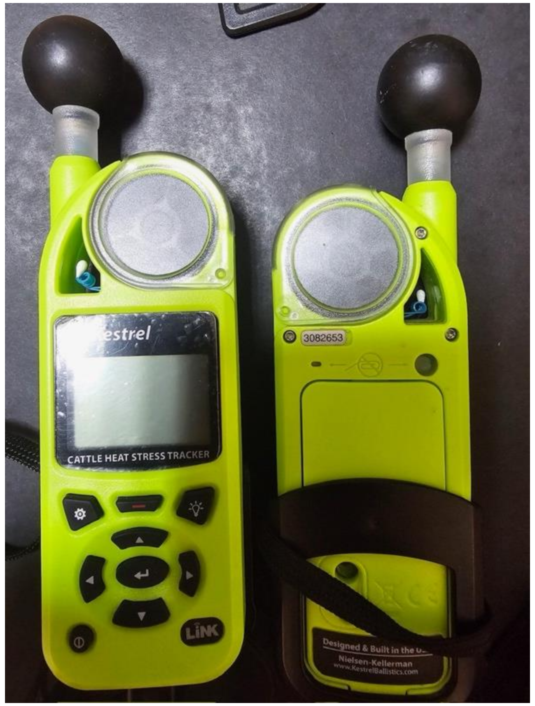


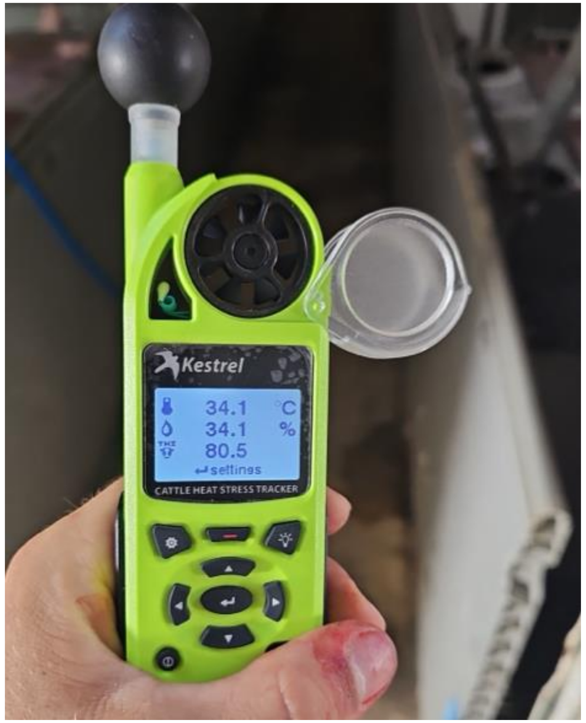


## Wireless Digital Weather Station Agenso MeteoIo T 2100S 4G (Agenso, Athens, Greece)

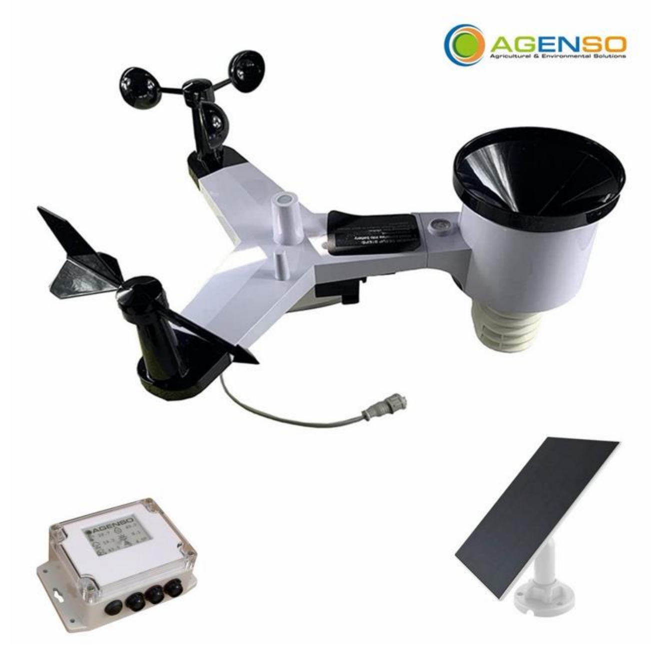


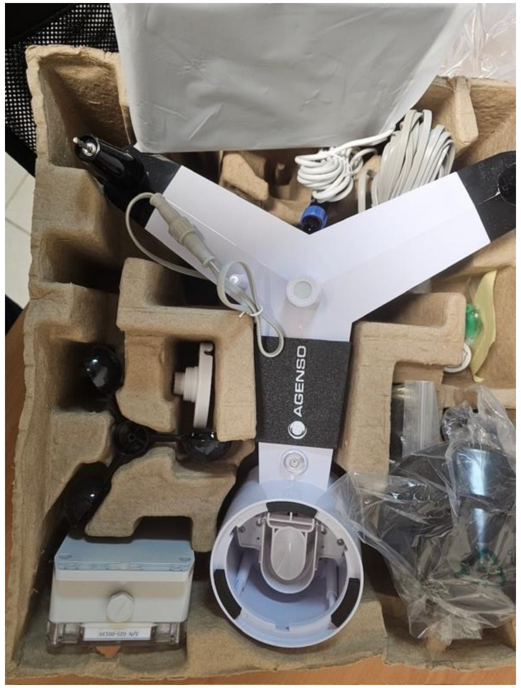

Supplement: Supplementary file 1 [file life-16-01306-s001.zip › Supplementary files _ R3/Supplementary File S3_ Equipment for THI monitoring .pdf]
