# Supplementary material for: Heat Stress, Indoor Microclimate, and Lactation Feed Intake in Commercial Mediterranean Sows: Parity Effects and a Daily Resolution Predictive Model
Source: Life (Basel). 2026 Aug 9;16(8):1306. doi: 10.3390/life16081306 (PMC13514657; doi:10.3390/life16081306)

# Trial farm – Farrowing house facilities

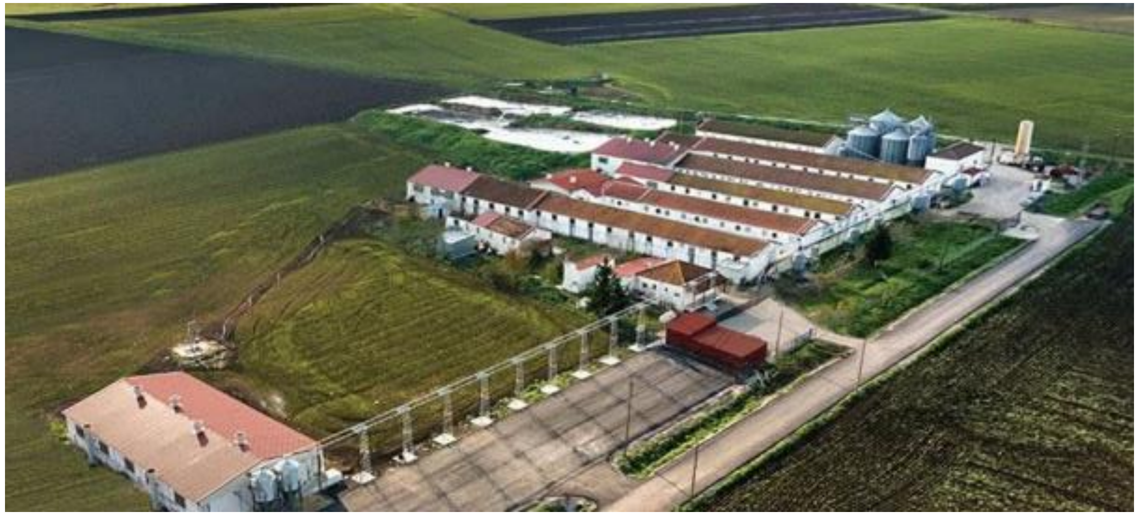

Automated GESTAL Solo+ feeding stations system (JYGA Technologies, St-Lambert-de-Lauzon, QC, Canada)

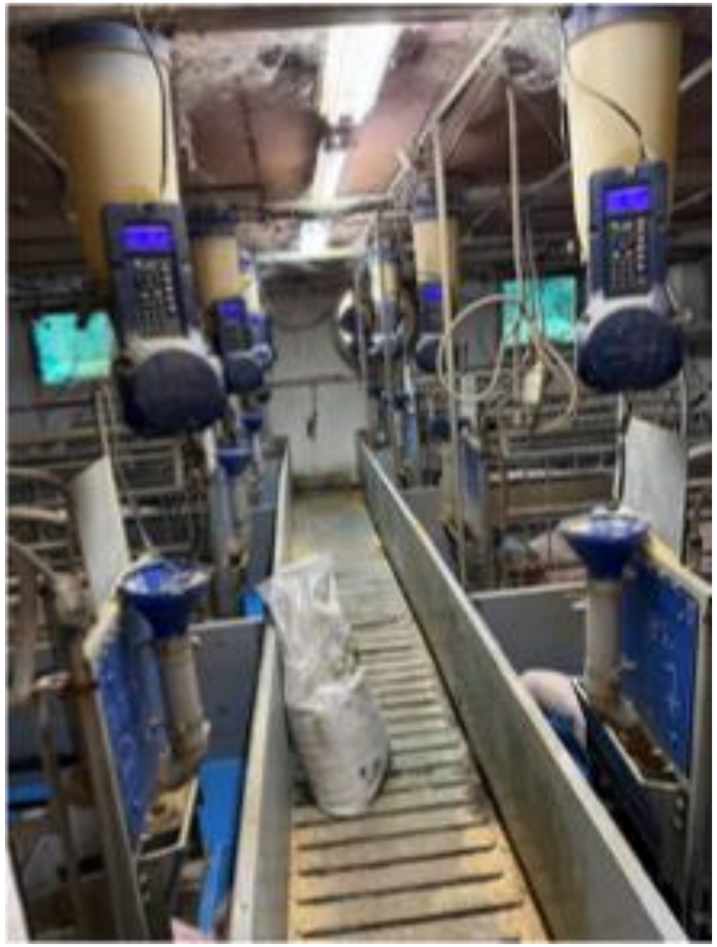

Automated d climate-control and ventilation management

(BlueControl Climate, SKOV A/S, Denmark)

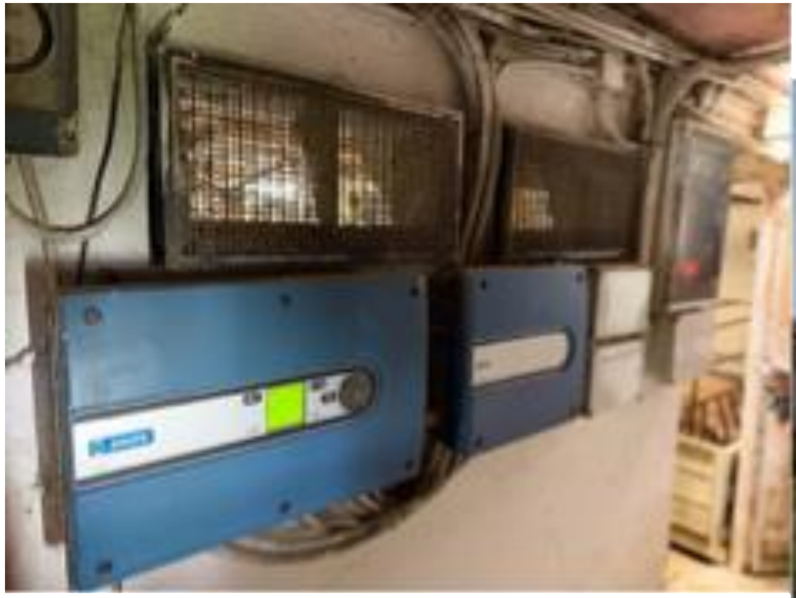

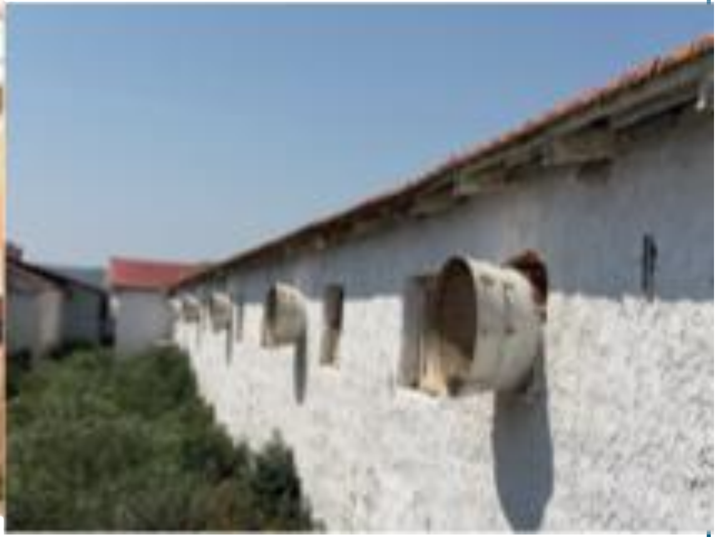

Supplement: Supplementary file 1 [file life-16-01306-s001.zip › Supplementary files _ R3/Supplementary File S2- Photos from the trial farm.pdf]
